# The Dietary Intake of Carrot-Derived Rhamnogalacturonan-I Accelerates and Augments the Innate Immune and Anti-Viral Interferon Response to Rhinovirus Infection and Reduces Duration and Severity of Symptoms in Humans in a Randomized Trial

**DOI:** 10.3390/nu13124395

**Published:** 2021-12-08

**Authors:** René Lutter, Annemarie Teitsma-Jansen, Esther Floris, Saeeda Lone-Latif, Abilash Ravi, Yanaika S. Sabogal Pineros, Tamara Dekker, Barbara Smids, Ridha Khurshid, Marcela Aparicio-Vergara, Rianne Ruijschop, Lara Ravanetti, Wim Calame, Alwine Kardinaal, Ruud Albers

**Affiliations:** 1 Amsterdam UMC, Department of Respiratory Medicine, University of Amsterdam, Meibergdreef 9, 1105 AZ Amsterdam, The Netherlands; s.j.lone@amsterdamumc.nl; 2 Amsterdam UMC, Department of Experimental Immunology, University of Amsterdam, and Amsterdam Infection & Immunity Institute, Meibergdreef 9, 1105 AZ Amsterdam, The Netherlands; a.m.teitsma-jansen@amsterdamumc.nl (A.T.-J.); a.ravi@lumc.nl (A.R.); y.s.sabogalpineros@amsterdamumc.nl (Y.S.S.P.); t.dekker@amsterdamumc.nl (T.D.); b.s.smids@amsterdamumc.nl (B.S.); ridha_55@msn.com (R.K.); l.ravanetti@amsterdamumc.nl (L.R.); 3 NIZO, Kernhemseweg 2, 6718 ZB Ede, The Netherlands; estherfloris@hotmail.com (E.F.); rianne.ruijschop@nizo.com (R.R.); alwine.kardinaal@nizo.com (A.K.); 4 NutriLeads B.V., Bronland 12-N, 6708 WH Wageningen, The Netherlands; Marcela.Aparicio@nutrileads.com (M.A.-V.); ruud.albers@nutrileads.com (R.A.); 5 StatistiCal B.V., Strandwal 148, 2241 MN Wassenaar, The Netherlands; w.calame@kpnplanet.nl

**Keywords:** anti-viral response, kinetics, rhinovirus-16, viral clearance, common cold, healthy adults, transcriptome

## Abstract

Acute respiratory infections are an important health concern. Traditionally, polysaccharide-enriched extracts from plants, containing immunomodulatory rhamnogalacturonan-I (RG-1), were used prophylactically. We established the effects of dietary supplementation with carrot-derived RG-I (cRG-I, 0–0.3–1.5 g/day) in 177 healthy individuals (18–65 years) on symptoms following infection with rhinovirus strain 16 (RV16). Primary outcomes were changes in severity and duration of symptoms, and viral load in nasal lavage. Secondary outcomes were changes in innate immune and anti-viral responses, reflected by CXCL10 and CXCL8 levels and cell differentials in nasal lavage. In a nested cohort, exploratory transcriptome analysis was conducted on nasal epithelium. Intake of cRG-I was safe, well-tolerated and accelerated local cellular and humoral innate immune responses induced by RV16 infection, with the strongest effects at 1.5 g/d. At 0.3 g/d, a faster interferon-induced response, induction of the key anti-viral gene *EIF2AK2*, faster viral clearance, and reduced symptom severity (−20%) and duration (−25%) were observed. Anti-viral responses, viral clearance and symptom scores at 1.5 g/d were in between those of 0 and 0.3 g/d, suggesting a negative feedback loop preventing excessive interferon responses. Dietary intake of cRG-I accelerated innate immune and antiviral responses, and reduced symptoms of an acute respiratory viral infection.

## 1. Introduction

Acute respiratory infections (ARI) are an important health concern and common colds alone account for 150 million workdays missed by employees in the US, with an economic impact of more than $20 billion annually [1]. ARI are also a major source of morbidity and mortality in patients with inflammatory airway disease, elderly and newborns [2,3]. They can condition airways for severe secondary infections [4], and emerging respiratory viruses can lead to life-threatening disease, as exemplified by influenza infections and recently by the SARS-Cov2 pandemic [5].

Most ARI resolve within one to two weeks and the host response is driven to a large extent by the innate immune system. Innate immune responses are triggered by signaling via pattern-recognition receptors (PRR) such as Toll-like receptors (TLR), double-strand RNA-activated protein kinase R (PKR) and retinoic acid-inducible gene-I (RIG-I)-like receptors that sense pathogen-associated molecular patterns (PAMPs) [6,7]. PRR funnel their signals into interactions with regulated signaling cascades, which then initiate the key antiviral interferon response as well as other common anti-viral and innate immune responses [8,9].

It is well known that deficiencies in protein energy, specific vitamins (A, B6, B12, folate, C, D and E) and trace elements (zinc, copper, selenium and iron) impair immune function [10,11], diminish responses to vaccination [12], and increase susceptibility to respiratory infections [13]. Other dietary components have a critical role in educating and regulating immune responsiveness and modulating microbiota composition and functionality in the gut [14,15]. Changes in the gut ecosystem can modulate respiratory immune responses and, inversely, respiratory infections can modulate the composition of the gut microbiota. This two-way dialogue, also described as the gut–lung axis (GLA), was shown in humans with recruitment of recirculating innate immune cells from the gut to the lungs and distant effects of short chain fatty acids produced by the gut microbiota [16].

Historically, specific plant extracts were used prophylactically for airway infections [17,18,19,20] and several randomized controlled trials have shown that daily intake (0.2 and 0.4 g/day) of polysaccharide-enriched ginseng extracts can modulate innate immune responses and reduce the incidence, severity and duration of natural viral respiratory infections [21,22,23,24,25,26]. Pectic polysaccharides and especially rhamnogalacturonan-I (RG-I) domains were identified as immunomodulatory constituents in ginseng and other plant extracts [27]. RG-I is a branched pectic polysaccharide comprising alternating α-1,2-L-rhamnose-α-1,4-D-galacturonic acid-units, substituted with arabinose and galactose side chains. Upon evaluating extracts from renewable sources, carrot rhamnogalacturonan-I (cRG-I) was found to very effectively modulate innate immune function [28], stimulate the production of short chain fatty acids by the microbiota [29], and increase innate immune cell function and enhance the efficacy of influenza vaccination in immunocompromised mice [30]. In humans, ex vivo immune responsiveness was dose-dependently enhanced and microbiota composition modulated after 4-week supplementation with a similar RG-I extract [28].

Challenging humans with the common cold virus rhinovirus-16 (RV16) causes only mild symptoms and is well-established as a model to study innate immune and anti-viral responses in concert with the development and resolution of symptoms [31]. Cold symptom severity can be rated based on a simple sum of severity points (0 denotes absent, more points for increasing severity) for various symptoms, e.g., sneezing, nasal obstruction, sore throat, etc., by using the Jackson score or the Wisconsin Upper Respiratory Symptom Score-21 (WURSS-21). Whereas the Jackson score defines and evaluates colds, the WURSS-21 questionnaire also includes questions related to cold-specific quality of life outcomes. The symptom scores for items 2–11 of the WURSS-21 questionnaire are commonly used to assess symptom severity and duration in clinical studies [32,33].

Based on the above, we postulated that the prophylactic use of cRG-I as a dietary supplement could enhance innate anti-viral responsiveness and consequently reduce symptoms of ARI in humans. In this randomized, double-blind, placebo-controlled trial, we investigated the effect of cRG-I at 0 g/day, 0.3 g/day (selected based on equipotency to 0.2 and 0.4 g/day ginseng dose in earlier studies) [21,22,23,24,25,26] and 1.5 g/day (arbitrarily selected to be 5 times higher), and we used a validated WURSS-21 questionnaire to evaluate the dose-effect of cRG-I on the symptom scores and duration of symptoms following a standardized ARI triggered by exposure to RV16 in healthy subjects. In a nested study, we used transcriptomics to explore the virus-induced local gene expression responses in nasal epithelial cells over time. In this study, cRG-I accelerated innate immune responses to RV16 in a linear dose-dependent manner. Whereas the high dose of cRG-I compared to no cRG-I triggered a faster interferon-induced response, remarkably, the intermediate dose more markedly reduced virus-induced symptoms in parallel with a significantly faster anti-viral interferon response. These findings are discussed in relationship to the complex regulation of interferon responses.

## 2. Materials and Methods

### 2.1. Study Design

**Figure 1:**
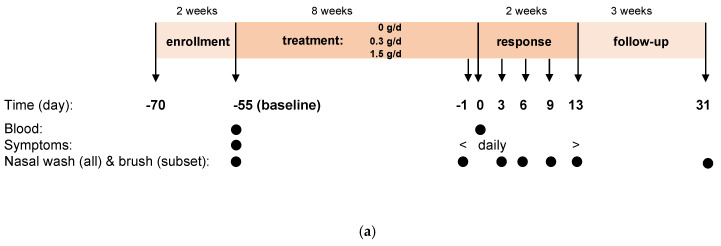

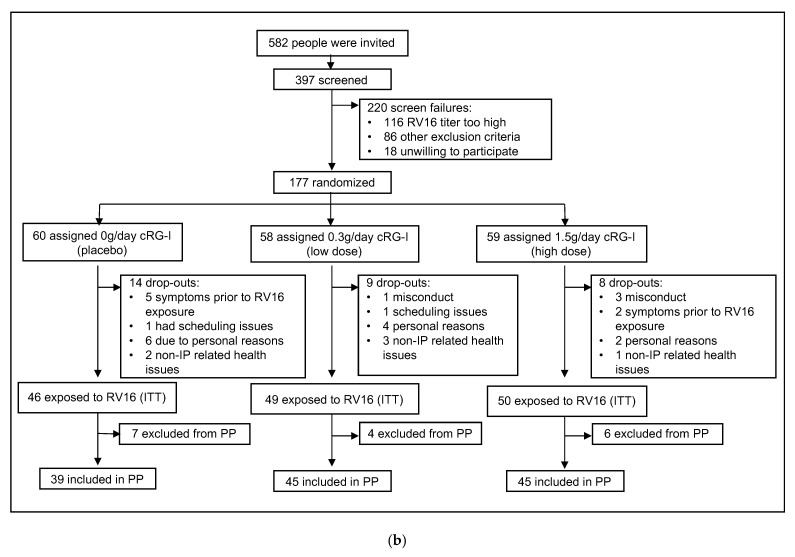
Study design (**a**) and disposition of subjects (**b**). Numbers indicate number of subjects. Unless indicated otherwise, results are indicated for all 146 subjects exposed to RV16 (ITT population).

This single center (Amsterdam University Medical Center, location Academic Medical Center, Amsterdam, The Netherlands), controlled, randomized, double-blind dose-response study comprised three arms with 0 (no-dose), 0.3 (low-dose) and 1.5 (high-dose) g/day in a parallel design. The study period had four phases: enrollment (screening, eligibility), treatment (8 weeks of study product intake), response phase (two weeks, exposure at day 0 (d0) to RV16 and course of infection) and follow-up (three weeks, safety parameters and seroconversion; Figure 1a). Each week, six subjects were block randomized by the pharmacy to the three arms (allocation ratio 2:2:2).

Subjects (free-living) completed an online WURSS-21 questionnaire on the day before infection (d-1) and subsequently every day until d13. Subjects also completed an online Jackson questionnaire from d-7 until d13, which was used to determine eligibility for the RV16 challenge. Nasal lavage was collected at baseline (d-55), just before infection (d-1) and after infection on d3, d6, d9 and d13. A throat swab on d-1 was used to detect natural viral infections by PCR and only symptom-free, PCR-negative subjects were exposed to RV16. In a nested subset (16 randomly selected participants per arm), nasal brushes were used to collect nasal epithelial samples for transcriptome analyses at the same timepoints as for nasal lavage [31]. Basic clinical chemistry was analyzed in blood samples collected at baseline (d-55), just before infection (d-1) and at the end of the study (d31). The first and latter samples were also used to determine anti-RV16 antibodies. Throughout the study, adverse events were noted and scored to assess any possible relationship to the food intervention.

A Data Safety Monitoring Board monitored safety data on an ad hoc basis and efficacy data when 50% of subjects had completed the study to provide recommendations for extension of the study or not.

### 2.2. Healthy Participants

Participants were assessed as healthy by the study physician based on medical history and medication use; they were between 18 and 65 years of age with a BMI between 18.5 and 30.0 kg/m^2^. Neutralizing antibodies against RV16 were determined in serum by 2-fold serial dilution after an initial 1 in 3 dilution as described [31], and participants with RV16 antibody titer >1:6 at screening were excluded. Volunteers with a medical history of hay fever, rhinosinusitis, asthma or COPD, any other underlying pulmonary, cardiovascular or auto-immune disease, or a food allergy were excluded. A complete overview of inclusion and exclusion criteria, the minimal dietary restrictions and other relevant criteria are provided in the Supplementary Material (Table S1).

### 2.3. Test Articles

cRG-I is a natural extract from carrot (*Daucus carota* subsp. *sativus*). The patented ingredient supplied by Nutrileads (Wageningen, The Netherlands) is a water-soluble non-digestible fermentable fiber enriched (80%) in the RG-I domain of pectin. The extraction method and extract characteristics (composition and structure) were described earlier [28]. The monosaccharide composition of cRG-I is (% mol/mol): 14.3 rhamnose; 34.8 arabinose; 19.6 galactose; 0.8 fucose; 4.3 glucose; 0.9 mannose; 0.7 xylose; 25 galacturonic acid. Test articles were prepared by mixing with maltodextrin and caramel color to obtain identical powders as follows: 0, 0.3 and 1.5 g cRG-I- extract, 3, 2.7 and 1.5 g Maltodextrin (MALDEX 170, Tereos, Belgium) and, for each dose, 0.5 g caramel color type 1 (Natural spices, Mijdrecht, The Netherlands) to obtain sachets with 3.5 g powdered supplement of identical volume and appearance for the no, low- and high-dose groups, respectively. Study products were to be taken with food and drink items of choice once a day with the first meal (preferably breakfast) during the treatment and response phase.

### 2.4. Procedures

The collection of nasal lavages is described in the online Supplementary Material. The lavage fluid was filtered on a cell strainer after which cells were separated by centrifugation (10 min at 465 g at room temperature (RT)) and processed for cell differentiation using a cytospin. The supernatant was aliquoted and stored at −80 °C, whereby an aliquot of 200 µL was stored for analysis of soluble mediators and similarly for determining the viral load by PCR for RV16 [31]. The soluble mediators were analyzed in multiplex by Luminex, apart from ECP and MPO, which were assessed on the MSD platform using electrochemiluminescence. Sequential samples were analyzed batch-wise to limit variability and internal controls were used to verify consistency. Participants were challenged by instillation of 100 median tissue-culture infectious doses (TCID50) RV16 (GMP-prepared RV16: UBiopred EU/IMI) in the nasal cavity [31]. Blood was sampled for safety monitoring and the clinical chemistry parameters included glucose (mol/L), ASAT (U/L), ALAT (U/L), eGFR, Gamma-GT (U/L), index bilirubin, index lipemia, creatinine (mol/L), immunoglobulins (g/L) and leukocytes, neutrophils, lymphocytes, monocytes, eosinophils and basophils (all #/L). Any significant changes between baseline (d-55) and just before exposure (d-1) in the analytes measured for safety monitoring were considered due to the study product.

### 2.5. Outcomes

RV16-exposed subjects were considered infected with RV16 if they had increased Jackson symptom scores and/or increased viral titers and/or increased RV16-specific antibody titers [31].

The primary outcome measures were symptom scores and duration of symptoms, using a validated Dutch translation of the standardized WURSS-21 on d-1 to d13, and the viral load by PCR in nasal lavage over 13 days following RV16 exposure [34]. The symptom scores for items 2–11 of the WURSS-21 questionnaire were used to assess symptom severity and duration.

Secondary parameters were the effect of cRG-I on duration of infection, based on the duration of RV16 viral titers in nasal lavage (measured on d0, d3, d6, d9 and d13) and WURSS-21 scores (measured daily from d0 to d13), and on change in CXCL10 (IP-10) and CXCL8 (IL-8) levels and cell differentials in nasal lavage (measured on d0, d3, d6, d9 and d13) following experimental infection. In the nested study, transcriptome analyses were conducted on nasal epithelial brushes collected before and after the treatment phase (d-55 and d-1) and during the response phase (d3, d6, d9, d13). Statistically significant dose-dependent effects of cRG-I on the primary and secondary outcome parameters were considered measures of efficacy. Analyses that were not prespecified as primary or secondary outcomes were considered exploratory.

### 2.6. Statistical Analyses

Prior data for a dietary intervention using an RV16 infection model were not available for a power calculation. Instead, for symptom scores, an effect size of 40% reduction in the area under the curve (AUC) of the WURSS-21 symptom scores, shown by others to be a relevant and achievable difference [32,33], was used and, for viral load, an arbitrary reduction of 10% in a single measurement was chosen [32,33,35,36]. For a one-sided difference with alpha 0.05 and correcting for the interim analysis, this would require 51 and 65 subjects per study arm for symptom scores and viral load, respectively. Making use of the variation per dose (*n* = 3 in total: 0–0.3–1.5 g/day) in one overall Generalized Estimating Equations (GEE) analysis could reduce the required number of subjects per group to 37 and 48, respectively [37]. Anticipating a drop-out rate of 15% led to 44 and 56 subjects per study group, respectively. Based on this, the study targeted an enrolment of 168 subjects. One-sided evaluation was performed since it was expected that consumption of the experimental compound would reduce both the WURSS score and viral load, as well as minimize the number of subjects that had to be infected with RV16.

The outcomes of symptom scores, duration and viral loads were analyzed using GEE, considering the different dose levels and all relevant time intervals, at and after infection. The absolute outcome per person per time interval per respective parameter was used as a dependent parameter. All other available factors, such as age, BMI, gender, etc., were used as independent parameters, and time and time-squared were also used to evaluate a potential parabolic fit in the respective outcome. Moreover, since time x dose was also accounted for in the GEE model, a potential difference in the time-dependent dose effect was also evaluated. Statistical analyses were conducted for both the intention-to-treat (ITT) and per-protocol (PP) population and sensitivity to outliers was tested applying the Grubbs’ test. The outcome for the PP population was similar to that for ITT, unless indicated otherwise.

The 50% score for reduction in severity of RV16 infection was estimated for the 0 g/day dose group, with and without repeated measurement, by using the interval showing the linear decrease (top of the curve—asymptotic lower outcome in the curve) in the WURSS scores (linearity was checked via GEE analysis). A similar procedure was followed for the 0.3 and 1.5 g/day doses to calculate the day at which the 0 g/day 50% reduction in the severity-score was established. Finally, the percentage reduction compared to the 0 g/d dose group was determined.

In some cases, a post-hoc analysis was performed to quantify a difference in outcome. This was also undertaken via GEE modelling prior to comparing two separate doses.

A *p*-value of 0.05 was considered to identify significance, with one-sided evaluation applied. Statistical analysis was performed via Stata, version 12 (Statacorp, College Station, Texas, USA), and GraphPad, version 6 (GraphPad Prism, San Diego, CA, USA); the latter was also used for graphical representation of the data. More detailed descriptions of the statistical analyses can be found in the Supplementary Material.

## 3. Results

### 3.1. Study Subjects

Three-hundred and ninety-seven participants of the 582 invited were screened, of whom 177 were enrolled in the study between July 2018 and May 2019 (60 in the no-dose group, 58 in the low-dose group and 59 in the high-dose group; Figure 1a,b).

Of the 177 randomized participants, 31 dropped out before RV16 exposure (14, 9 and 8 in the no-, low- and high-dose groups, respectively) mainly due to concomitant natural infections. The interim analysis did not lead to any change of the original protocol. Characteristics of the 146 subjects in the RV16-exposed ITT population are presented in Table 1.

This was not different from those of the 177 enrolled subjects or the 48 subjects in the nested sub study (Supplementary Table S2). Overall compliance in terms of daily intake of the test article (99%) and adherence to protocol were excellent. During the blind data review, 17 subjects were excluded from the PP population for either major protocol deviations (non-compliance with investigational product (IP)-intake (1), non-compliance WURSS-21 completion (2), suspected of pre-challenge viral infection (5), inadvertent medication use (1), IP use outside acceptable timeframe (1)) or because they did not show any objective signs of respiratory infection, based on the Jackson score and viral titer in the 14 days following exposure to RV16, as well as the RV16 antibody titer on d31 (in total 7 subjects; 1, 3 and 3 subjects in the no-, low- and high-dose groups, respectively).

### 3.2. Safety and Tolerability

Intake of the study product from d-55 to d-1 did not negatively affect clinical chemistry. In fact, despite high variation, there was a significant direct cRG-I-dose-related reduction for ALAT (*p* < 0.009), ASAT (*p* < 0.002), GGT (*p* < 0.03), and the bilirubin (*p* < 0.01) index after adjusting for potential confounders (age, gender, BMI, alcohol consumption and vegetarianism), see Table S3 in the Supplementary Material.

cRG-I was well tolerated, which was reflected by the lack of differences in severity and frequency of adverse events including those with a possible relation to the study product. In 402 separate registrations, 432 adverse events (AEs) were recorded, with 144, 149 and 139 in the no-, low- and high-dose groups, respectively. Sixteen AEs were scored as possibly related to study product intake, but there was no relationship between either group and the incidence of AEs, before or after RV16 exposure. The only reported severe adverse event transpired to be a pregnancy. The baby was born in good health and progressed well during one-month follow-up.

### 3.3. Effect of cRG-I on Symptom Scores

**Figure 2:**
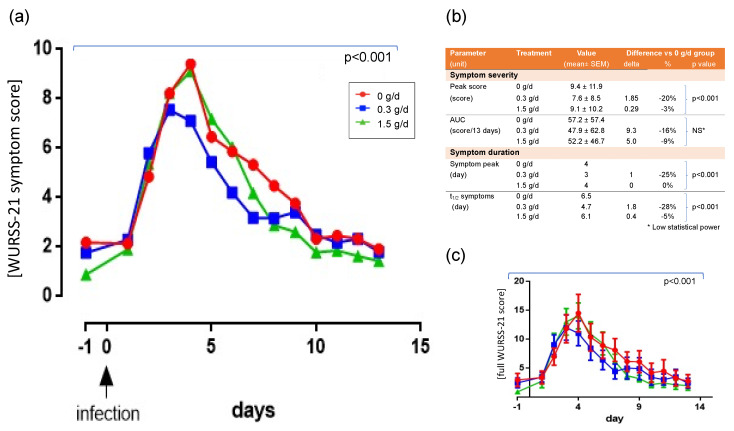
Perceived symptoms following infection with RV16. Symptom score (primary outcome, item 2-11 of WURSS-21 questionnaire, shown without error bars for clarity). The GEE model (fit established via Wald Chi square: 1480.72; *p* < 0.0001) showed a parabolic association in time as well as dose of cRG-I. Analyses showed a highly significant shift of the curve to the left (reduction in duration) and lower maximum (reduction in severity) over the 13 days following infection. These effects were parabolically associated, with the dose of cRG-I being very apparent in the 0.3 g/day group whereas the 1.5 g/day group was similar to the no-dose group (**a**). Quantification of effects on symptom severity using peak score and AUC, and on duration using symptom peak day and pharmacokinetic modelling of the t1/2 symptoms/day; * low statistical power (**b**). Full WURSS-21 score including the quality-of-life items, shown with SEM to illustrate variability between subjects. The GEE model (fit 2681.77; *p* < 0.0001) yielded a similar parabolic dose-dependent outcome: *p* < 0.001, indicating that for the 0.3 g/day group, the full WURSS-21 score and also the peak score was lower and reduced earlier. Post-hoc quantification of effect sizes for the 0.3 and 1.5 g/day groups indicated a reduction in symptom score height of 22% and −0.1%, and a reduction in peak response/day of 14% and 0.3%, respectively (**c**).

Results for the ITT and PP populations were similar; data for ITT are presented unless indicated otherwise. The WURSS-21 symptom scores showed time-dependent parabolic curves for all three doses (GEE model coefficient −0.03 (5% CI interval: −0.03–−0.02)) for WURSS items 2–11. The outcome revealed an earlier decline of symptoms and less severe symptoms in the low-dose group, compared to the other groups. The average peak symptom score was observed on d3 in the low-dose group and on d4 for the no and high-dose groups (Figure 2a). Despite individual variability in perception of symptoms, this 25% earlier onset of decline of symptoms in the low-dose group was statistically significant (*p* < 0.001), whereas the rate of decline (slope of the downward curve) was similar for all groups. Post-hoc analysis using a pharmacokinetic model indicated that symptoms reduced to 50% (“symptom elimination half-life”, t_1/2_) at day 6.5 in the no-dose group, whereas this was at day 4.7 (−28%) for the low-dose group and day 6.1 (−5%) for the high-dose group (Figure 2b). There was also a 20% reduction in peak symptom scores in the low-dose group (7.6 ± 8.5) compared to other groups (no-dose: 9.4 ± 11.9; high-dose: 9.1 ± 10.2; *p* < 0.001). In summary, there was a parabolic dose dependency (GEE model coefficient 0.75 (95% CI interval: 0.47–1.03)) and the dose of 0.3 g/day was most effective in minimizing the total symptom score in both severity (height) and duration (time, GEE model coefficient: −0.005 (95% CI interval: −0.007–−0.004)). Similar findings were obtained using data from the complete WURSS-21 questionnaire including the quality-of-life questions (Figure 2c). In line with these findings, the AUC of the symptom scores (−16%, Figure 2b) and duration of infection based on symptoms (Supplementary Figure S1b) were also reduced in the low dose group, but these did not reach significance due to a loss of power after reducing the kinetic data to a single data point. There were no significant differences in viral load between groups (Supplementary Figure S1a). Looking at specific intervals (post-hoc analysis), the increase in log_10_ viral load as assessed by PCR in lavage between d1 to d3 was the highest (*p* < 0.02) for the low-dose group compared to the other groups.

### 3.4. Effect of cRG-I on Local Innate Immune Response

**Figure 3:**
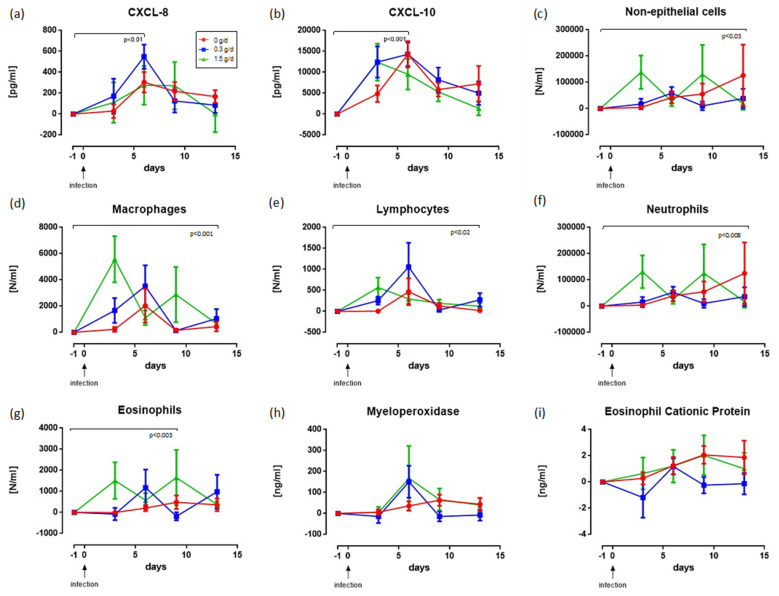
Time-dependent changes in CXCL-8 (**a**), CXCL-10 (**b**), cells in nasal lavage fluid (**c**–**g**) and granular proteins (**h**,**i**) expressed as change versus value just prior to infection (d-1). Each symbol represents the mean ± SEM. Significance was evaluated by step wise application of the change values of observations per person between d-1 and d13. The reported *p*-value in the various figures indicates the observed dose-dependent effect over the maximal time after infection for which significance was observed. (**a**) A dose of 0.3 g/day showed an earlier increase in values than doses of 0 and 1.5 g/day, and a higher peak than a dose of 0. (**b**) Higher doses led to earlier responses. (**c**) Higher doses led to higher numbers of cells in a time-dependent (*p* < 0.03) parabolic fashion. (**d**) Higher doses led to higher numbers of cells in a time-dependent (*p* < 0.001) parabolic fashion (**e**) A dose of 0.3 g/day yielded higher numbers of cells than doses of 0 and 1.5 in a time-dependent (*p* < 0.002) parabolic fashion. (**f**) Higher doses led to higher numbers of cells in a time-dependent (*p* < 0.08) parabolic fashion. (**g**) Higher doses led to higher numbers of cells in a time-dependent (*p* < 0.003) parabolic fashion. (**h**) No significant difference in outcome was found between doses. (**i**) No significant difference in outcome was observed between doses. The cellular data (panels **c**–**g**) above show relatively large standard errors of the mean due to some outliers affecting the mean profiles, especially on d9 and d13. Supplementary Figure S3 shows the same graph with the highest and lowest outliers removed; Supplementary Figure S2 shows the absolute values including the values at baseline (d-55).

Analyses of nasal lavage for markers of the local innate immune response indicated no significant differences due to cRG-I prior to RV16 exposure (d-55 compared to d-1) (Supplementary Figure S2). However, GEE analyses revealed a marked acceleration of the infection-induced responses measured as the amount of CXCL-8 and CXCL-10 on d3 and d6 (Figure 3a,b). The influx of non-epithelial cells, in particular neutrophils, macrophages, lymphocytes, and eosinophils (Figure 3c,g), was also enhanced with responses generally being fastest/highest in the high-dose cRG-I group, except for the influx of macrophages and lymphocytes, which were highest for d6 in the 0.3 g/day group when their numbers were already decreasing in the 1.5 g/day group. Cell numbers in the nasal lavage were affected by a few outliers, as can be seen by the large SEM at several time points. Supplementary Figure S3 shows the same graphs with these outliers removed. Changes in myeloperoxidase (MPO; Figure 3h) and eosinophil cationic protein (ECP; Figure 3i) parallel changes seen in neutrophil and eosinophil cell counts, respectively (trend only). These differences in initial local innate immune responsiveness did not affect the induction of subsequent humoral adaptive immunity as antibody titers to RV16 in blood taken 31 days after infection were not different between treatment groups (Supplementary Figure S1c).

### 3.5. Effect of cRG-I on Expression of Critical Interferon Response Genes in Nasal Epithelium

**Figure 4:**
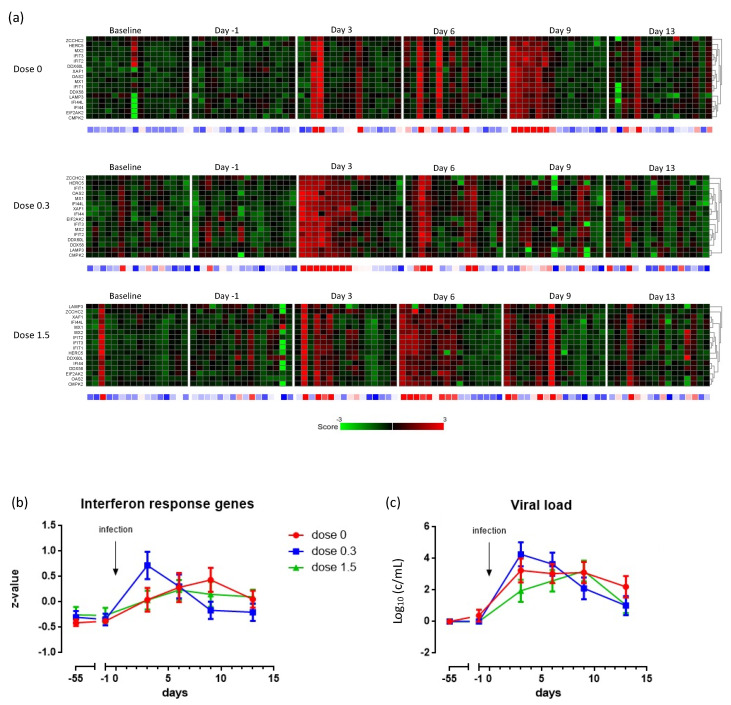
Summary of nested study with 16 subjects per group from whom nasal brushes were collected. Heatmaps of interferon-induced genes in nasal epithelial cells over time and as a function of the treatment (**a**). Baseline is prior to intervention; other time points are relative to the infection with RV16 (=d0; see Figure 1). Each checkerboard summarizes the data for 16 subjects of a group at a specific time point. Columns represent individual subjects (always in same order) and rows represent individual interferon response genes. In the heatmap: red and green represent up- and down-regulated genes, respectively. Black represents unchanged expression. The row below the heatmaps corresponds to total z-scores for each subject, where gradations of red indicate an increased transcription level and gradations of blue indicate a decreased level of transcription. The total z-scores (±SEM) over time as a function of the dose (**b**), and the viral load in the nasal lavage over time, as a function of the dose (**c**), are shown.

Global comparison of the transcriptome of nasal epithelial cells indicated that on d3, and especially d6, more genes were differentially expressed in the low dose group than in the two other groups and, on d13, the high dose group had the highest number of differentially expressed genes (Supplementary Table S4). Zooming in on critical interferon response genes, the heatmaps and GEE analyses of their combined z-score (Figure 4a,b) showed that, despite heterogeneity in timing between individuals, there was a highly significant interferon-induced response already on d3 in the low-dose group (*p* < 0.001), and on d9 and d6 for the no- and high-dose group, respectively. In addition to an earlier interferon-induced response, the genes *EIF2AK2* and *ZCCHC2* were expressed uniquely in the low-dose group (Supplementary Figure S4). Moreover, the GEE analyses demonstrated a higher initial load of viral RNA in the nasal lavage on d3 (*p* < 0.001) and a more rapid clearance (*p* < 0.001) in the low-dose group of the nested sub study (Figure 4c).

## 4. Discussion

This is the first paper to describe a protective effect of cRG-I, given as a dietary supplement, in a randomized, double-blind study, on common cold symptoms, particularly in terms of the acceleration of innate immune responses triggered by an experimental RV16 infection. This standardized RV16 challenge model provided the unique opportunity to investigate, in humans, the effect of cRG-I on the kinetics of early innate immune and anti-viral responses in conjunction with the perceived impact on symptoms using a validated questionnaire. Compliance in the study was high, cRG-I was well tolerated, and despite variability in individual responsiveness, the results were remarkably coherent and robust between outcomes and between the ITT, PP and nested subset populations.

The prophylactic intake of low-dose (0.3 g/day) cRG-I, which was selected based on earlier studies with ginseng, significantly reduced the severity of cold symptoms by 20%, and their duration by 25%, compared to no-dose (placebo) and the arbitrarily selected high-dose administration. The low-dose cRG-I also induced a faster clearance of virus than the no- and high-dose administration and showed that innate and anti-viral responses in humans can be accelerated by dietary supplementation with cRG-I. In the nested study, the intake of 0.3 g/day cRG-I resulted in an accelerated innate interferon response and enhanced expression of the *EIF2AK2* and *ZCCHC2* genes in infected nasal epithelial cells. These findings are in line with the reduction in symptoms from seasonal respiratory infections following supplementation with 0.2 to 0.4 g/day ginseng-derived polysaccharides [21,22,23,24,25,26] comprising RG-I [28], and with enhanced production of interferon gamma in response to an influenza vaccine in immunocompromised mice supplemented with carrot RG-I [30]. As in our study, the interferon response in mice only became evident after a challenge, i.e., delivery of the vaccine.

The dose–response relationships found in the current study are remarkable in that the acceleration and enhancement of the initial innate immune response, characterized by increases in CXCL-8, CXCL-10 and the infiltration of cells into the nasal lavage, was generally more pronounced with higher doses of cRG-I. This is in line with the dose-dependent enhancement of ex vivo innate immune function by a similar RG-I preparation in a human proof of concept study [28]. In contrast, the accelerated induction of interferon response genes showed a parabolic dose–response relationship with the fastest induction in the 0.3 g/day group, similar to the faster viral clearance and the unique induction of the *EIF2AK2* and *ZCCHC2* genes. These responses also correspond to the parabolic dose–response relationship observed with reduced symptom scores. Such bell-shaped dose–responses are not unusual and have also been shown for vitamin E supplementation and the response to vaccination [38].

The accelerated interferon response peaking on d3 in the low dose cRG-I group appears to be crucial for faster clearance of the virus and reduction in symptom scores. Interferon production is known to be strictly regulated, with the involvement of negative feedback loops [39]. This, combined with the observation that infiltration is already maximal on d3 (the first sampling point after infection), leads us to speculate that the high-dose cRG-I-induced high interferon production prior to d3, via, e.g., recruitment of more (responsive/active) macrophages, which may have resulted in the temporal down-regulation of the interferon response in the high-dose group, possibly explaining the bell-shaped dose–response curve and suggesting that the pathways involved are (differentially) regulated, possibly to prevent excessive responsiveness. As we compare response kinetics between groups, it is not clear from our data whether the interferon-induced response was accelerated for each individual participant, or for some participants. Furthermore, differences in antiviral responses and symptoms observed in the present study seem to arise very early after RV16 exposure, possibly as early as day 1 or 2; these aspects should be investigated in more detail in future studies.

The exact function of the *ZCCHC2* zinc finger protein is unknown; however, some of the zinc finger family of proteins are known to play a key role in antiviral responses [40,41]. *EIF2AK2* encodes Protein Kinase R (PKR), which is a crucial innate antiviral protein that senses viral replication and shuts off host protein synthesis, thereby limiting viral replication [42]. We speculate that the qualitative differences observed in the interferon response between high- and low-dose cRG-I, as exemplified by *ZCCHC2* and *EIF2AK2* gene expression, likely contributes to the antiviral effects of low dose cRG-I. This is in line with the accelerated clearance of RV16 in the low-dose group in the nested study and the reduced symptoms in the ITT population. Trends in viral loads in the ITT population (Supplementary Figure S1a) concur with the findings in the nested study.

Even though the exact mode of action of cRG-I is still under investigation, a dual mechanism was proposed [15]. Firstly, cRG-I passes the upper gastrointestinal tract (GIT) intact, and it was postulated that dendritic cells and macrophages surveilling the intestinal tract sense cRG-I by sampling from the lumen or in Peyers Patches [43]. This would result in priming or training of these cells, which, after recirculation to the respiratory tract, alters local responsiveness to a respiratory infection. RG-I pectins have been shown to interact with TLR [15,27,44] and trained immunity was provided as an explanation in the immunocompromised mouse study where a preparation similar to cRG-I was found to enhance innate immunity and increase the efficacy of an influenza vaccine [30]. Secondly, it was shown that cRG-I is fermented in the lower GIT, affecting the composition and function of the gut microbiota [29] which are crucial factors modulating immune responses to vaccines [12] and infections, for example, by generating biologically active metabolites such as short chain fatty acids [14,45]. Detailed analyses of the fecal samples collected in the current study, and further mechanistic studies, are required to clarify the mode of action.

The innate immune system tends to channel responses to respiratory viruses through a limited set of signaling cascades, and our data indicate that both the timing and the extent of the interferon response are critical determinants in shaping the course of the infection. In a recent RV16 challenge study with asthma patients, interferon responses were also shown to vary in timing and extent between individuals, and were linked with viral clearance and symptoms [31]. The extent to which our data can be extrapolated to the protective effects of cRG-I in other populations or for other respiratory viral infections remains to be determined.

## 5. Conclusions

In conclusion, this randomized, double-blind, placebo-controlled study demonstrated that a dietary intervention with cRG-I had clinically significant effects by attenuating the severity and duration of symptoms in an experimental rhinovirus infection in humans. Innate immune responses were accelerated, and transcriptome data showed an early interferon-induced response with EIF2AK2 and ZCCHC2 genes uniquely expressed in the low-dose group. To the best of our knowledge, this is one of the most effective interventions shown in the RV16 challenge model [35,46,47,48,49,50]. Given that the initial response to respiratory virus infections relies heavily on common innate antiviral and immune responses, cRG-I intake may also prove effective for limiting other respiratory viral infections. RG-I from carrot appears to be an efficacious solution that is safe, sustainable, affordable and could easily be integrated into food products or dietary supplements that aim to enhance protective innate immune responsiveness.

## Figures and Tables

**Table 1:** Demographic characteristics of all subjects exposed to RV16 (ITT population *n* = 146).

Parameter	Statistic	0 g/Day	0.3 g/Day	1.5 g/Day	*p*-Value *
N		46	49	51	
Age (y)	Mean ± SD	38.2 ± 15.8	35.4 ± 14.4	34.5 ± 14.9	0.46
Male (%)	N (%)	10 (22)	10 (20)	9 (18)	0.88
BMI (kg/m^2^)	Mean ± SD	23.2 ± 2.6	23.5 ± 2.9	23.8 ± 2.7	0.58
Alcohol consumption (glasses/week)	Median (range)	2 (0–10)	1 (0–12)	0 (0–10)	0.68
Vegetarian diet (N)	Median (range)	0 (0–1)	0 (0–1)	0 (0–1)	0.98

* Baseline differences over treatment groups were tested with ANOVA (age and BMI) or Kruskal–Wallis (gender, alcohol use, vegetarian diet). Characteristics of enrolled population and nested subset as well as details on inclusion, exclusion (e.g., smoking, allergies and underlying lung diseases) and restriction criteria are provided in the online data in the Supplementary Materials.

## Data Availability

The data presented in this study are available on any reasonable request from the corresponding author. The data may become publicly available at a later stage when technical quality of the data can be maintained and when time permits.

## Supplementary Materials

The following are available online at https://www.mdpi.com/article/10.3390/nu13124395/s1, Supplementary Methods, Figure S1: (**a**) RV16 RNA titer in NAL, (**b**) duration of infection, (**c**) RV16 antibody titer in blood, Figure S2: Absolute values for soluble markers and cells subsets in nasal lavage, Figure S3: Changes in soluble markers and cell subsets in nasal lavage without highest and lowest outliers, Figure S4: Expression of *EIF2AK2* and *ZCCHC2* genes in nasal epithelial cells, Table S1: Inclusion and exclusion criteria, Table S2: Demographic characteristics of enrolled subjects and nested subset, Table S3: Clinical chemistry, Table S4: Number of differentially expressed genes in brushed nasal epithelial cells compared to expression just prior to RV16 exposure.
