# Supplementary material for: The Dietary Intake of Carrot-Derived Rhamnogalacturonan-I Accelerates and Augments the Innate Immune and Anti-Viral Interferon Response to Rhinovirus Infection and Reduces Duration and Severity of Symptoms in Humans in a Randomized Trial
Source: Nutrients. 2021 Dec 8;13(12):4395. doi: 10.3390/nu13124395 (PMC8704532; doi:10.3390/nu13124395)
Supplement: Supplementary file 1 [file nutrients-13-04395-s001.zip › nutrients-1467603-supplementary.pdf]

Supplementary material

The dietary intake of carrot-derived rhamnogalacturonan-I accelerates and augments the innate immune and anti-viral interferon response to rhinovirus infection and reduces duration and severity of symptoms in humans in a randomized trial

First author René Lutter

Supplementary methods:

Study Procedures

For collection of nasal lavage, the nostril was closed off using an inflatable catheter (Medeco: Curion CuriStay catheter, latex 2-way, article nr 822116). To that end, the balloon was inflated with a 10 ml syringe (6-10 ml air), and another 10 ml syringe prefilled with warm (37°C) 10 ml of sterile 0.9% sodium chloride is attached to the shortened opening of the nasal adapter. Participants were seated in a 'writing' position with the head slightly tilted to the side of the catheter. Slowly, 10 ml of sterile PBS or HBSS was instilled via the catheter and left for 5 minutes. The fluid was withdrawn into the syringe and flushed back into the nasal cavity twice and nasal fluid was collected in a pre-weighed 15 ml polypropylene tube for processing. The total volume of lavage recovered was recorded. Participants were not allowed to blow their nose during this procedure. Straight after collection, tubes were transferred to the lab. Nasal lavage fluid was filtered on a cell strainer after which cells were separated by centrifugation (10 mins at 465g at room temperature (RT) and processed for cell differentiation using a cytospin). The supernatant was aliquoted and stored at -80°C, whereby an aliquot of 200 µL was stored for analysis of soluble mediators and similarly for determining viral RNA by PCR for RV16 (1).

Randomization and masking

Upon consent and eligibility, participants were allocated a unique identification number. Six participants per week were assigned to one of three treatment groups according to a double-blind randomization (2:2:2) schedule. Simple randomization was done manually by trained pharmacy staff assigning the participant identification number to the next available

randomization number on the randomization list. For each treatment 5 complex production codes were generated, and participants received one production code throughout the study. Members of the study team remained blinded till after a blind data review meeting.

RNA isolation from nasal brushes

The nasal brushes, which consisted predominantly of nasal epithelial cells, were obtained with a brush (1). The predominance of nasal epithelial cells was verified by the absence of specific markers for eosinophils (eosinophil cationic protein), neutrophils (myeloperoxidase) and macrophages (CD206) in the transcriptome of these brushings. Two brushes were pooled for each sample and centrifuged at 1000g for 10 mins at 4°C (Rotanta 460S, brake 3). The pellet was dissolved in 1 ml of TRIzol™ and stored at -80°C until RNA was isolated. After all samples were obtained, they were thawed at RT and, after 200 µl of chloroform was added, shaken vigorously for 30 seconds. The samples were kept at RT for 10 mins and then centrifuged at 16,000g for 15 mins at 4°C. The aqueous phase was cleaned up and concentrated with protocol 5.3 using the Nucleospin® RNA XS extraction kit (Macherey-Nagel). The quality and concentration of the samples were assessed by fragment analyzer (Advanced Analytical Technologies, Inc.).

cDNA preparation and RNA sequencing

The NEBNext Ultra Directional RNA library prep kit for Illumina was used to process the samples. The sample preparation was performed according to the protocol "NEBNext Ultra Directional RNA Library Prep Kit for Illumina" (NEB #7420S/L). Briefly, oligo-dT magnetic beads were used to isolate mRNA from total RNA. cDNA synthesis was performed after fragmentation of this mRNA. This was used for ligation with sequencing adapters followed by PCR amplification of the resulting product. The quality and yield after sample preparation was measured with the fragment analyzer. The size of the resulting products was consistent with the expected size distribution between 300-500 base pairs. To evaluate the quality of the library preparation and kits used, the raw data was sampled and mapped to annotated genomic references. Mapping positions were classified as intragenic, exonic, intergenic, intronic and rRNA. Clustering and DNA sequencing was performed according to the manufacturer's protocol using the Illumina NextSeq 500. A concentration of 1.6pM was used as input. The reads were trimmed for adapter sequences using Trimmomatic v0.30 before the alignment. Presumed adapter sequences were removed from the read when the bases matched a sequence in the adapter sequence set (TruSeq adapters) with 2 or less mismatches and an alignment score

of at least 12. The reference of ‘Homo sapiens. GRCh37.75’ was used to align the reads. The mapping to the reference sequence was done using a short reader aligner based on Burrows-Wheeler transform. The default of mismatch rate of 2% (3 mismatches in a read of 150 bases) was used.

Sequence data analysis

The frequency of the reads mapped on the transcript was determined as counts, which is used as an input for downstream analysis. Additionally, RPKM and FPKM (reads/fragments per kilobase of exon per million reads mapped) values were calculated. The read counts were loaded into the DESeq2 package, a statistical package within the R/BioConductor platform to determine the differentially expressed genes (paired). Image analysis, base calling and quality check was performed with Illumina data analysis pipeline RTA v2.4.11 and Bcl2fastq v2.17.

The z score for each gene is calculated by subtracting the mean interferon response gene expression of all individuals from the gene expression of the individual and divided by the standard deviation. For each individual the total z score (the row below the heatmaps) is calculated by sum of the z scores for all genes in the gene set and divided the result by the square-root of the total number of genes.

Statistical analysis

During a blind data review meeting, the study team decided on subject inclusion in the PP population based on protocol deviations as well as on missing and outlying data points. After this meeting the statistical analyses were completed, and the study was unblinded. Firstly, a comparison of the baseline variables in the three groups was performed using an unpaired parameter-free Kruskal-Wallis rank test or the unpaired parametric ANOVA test, depending on the distribution of the parameter investigated. The latter was evaluated applying the Shapiro-Wilk test.

For the impact of dose on the (absolute) WURSS scores (time interval d-1 till d13) a repeated measures General Estimating Equations (GEE) non-linear model was used, in a stepwise fashion in an individual-repeated design. The model used the WURSS score outcome as dependent parameter and as independent parameters various confounding factors, such as gender, BMI, infected (yes *vs*. no), alcohol consumption, sporting activities and vegetarian diet intake and start value of the respective dependent parameter, but most importantly time (in days), dose (0 – 0.3 – 1.5 g/d) and interaction terms between time and dose. The fit of the model was evaluated via Wald Chi square test, only when the fit showed a significant explanation of

the variation covered, was the outcome of the GEE model considered statistically relevant. The dose-dependent effect size per dependent parameter was expressed as coefficient in the model including the 95% Confidence Interval. Since in the model a parabolic association in time and dose was also addressed, the outcome focused on peak levels of both time and dose as well. For all other parameters (time interval d-1, d3, d6, d9, d13) the same procedure was followed during which change values were used as dependent parameters. The changes were calculated by subtracting the value obtained at d-1 from those at all intervals, leading to “0” at d-1. Discrete values were analyzed with a Poisson whereas continuous data with Gaussian distribution, the latter after checking normal distribution and normality in the residual fraction. For dose-dependent effects of cRG-I, GEE analyses were performed as described above.

When GEE was not feasible the parametric ANOVA and/or parametric-free Kruskal-Wallis test were/was applied. This approach was also used to detect potential time-dependent effects by the study product on various parameters (by analysing the outcome of specific time intervals), the z-values of the interferon-induced genes and the subset of the viral load data.

A pharmacokinetic model (post-hoc analysis) was used to evaluate differences in efficacy of reducing the symptoms in the WURSS score. From the outcome of the symptom score in the WURSS questionnaire, as obtained for all doses, the scores reached during the time interval between the absolute maximum of score (peak score) to the asymptotic outcome after having reached this maximum was selected to mathematically calculate the time to reach the 50% reduction score in dose “0” (no-dose group). Linearity of this interval was checked via GEE analysis and this 50% score was calculated per person from the linear regression line (decreasing in time) of the no-dose group. Subsequently, the (decreasing) regression lines over the same interval (peak value to asymptotic outcome afterwards) were determined for doses 0.3 and 1.5 g/day. The 50% reduction value for the no-dose group was used in the regression lines for doses 0.3 and 1.5 g/d to determine the day at which exactly that score was established. Finally, the percentage reduction or increase relative to the no-dose group was established.

Statistical analyses were performed using STATA, version 12.1 (StatCorp, College Station, Texas, USA) and GraphPad, version 6 (GraphPad Prism, LaJolla, CA, USA), the latter was also applied for graphical display.

Supplementary Results:

Comparison of baseline characteristics of the enrolled population and the nested study set showed that these were not different from the ITT population and that with exception of alcohol use in the nested sub study did not differ between subjects (supplemental Table S2).

Comparison of the transcriptome of nasal epithelial cells before (d-55) and after 8 weeks of intake (d-1) of cRG-I did not reveal differentially expressed genes significantly associated with gene sets. The response to RV16 infection compared to d-1 (Table S3) showed more immediate and pronounced responses (gene sets in brackets) at d3 and d6 in low-dose group (interferon response genes), whereas that in the no-dose group was delayed till d6 (inflammatory response genes) and d9 (interferon response genes) and even more so in the high-dose group at d9 (interferon response genes) and d13 (inflammatory response genes).

Supplementary Figures:

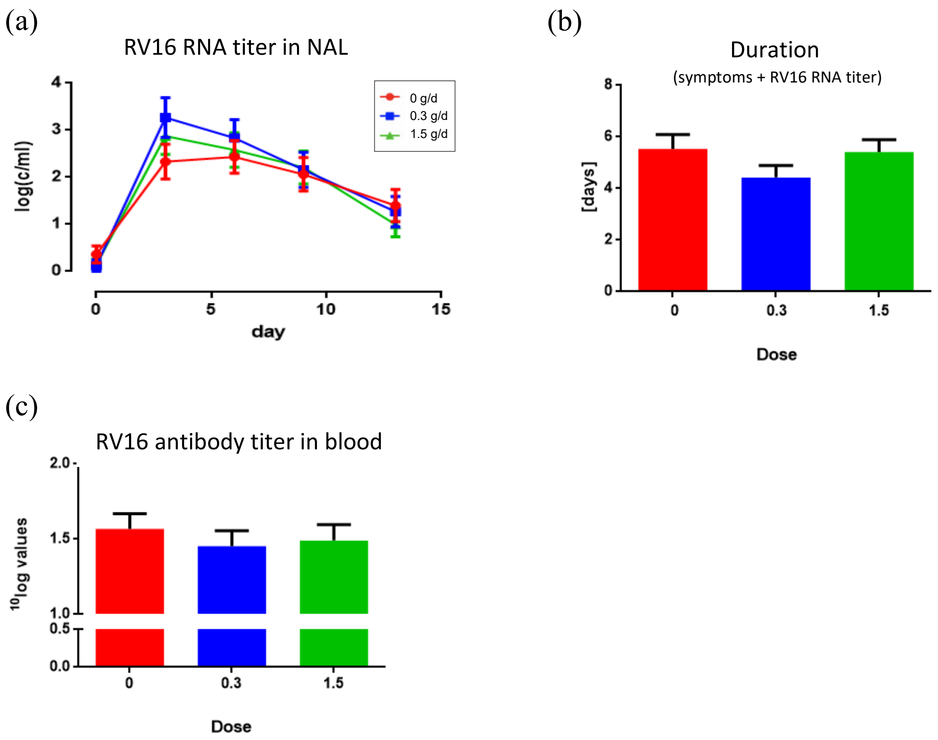

Supplementary figure S1: The RV16 viral load measured as PCR titer in nasal lavage fluid after $\log_{10}$ transformation (a), the duration of infection based on a composite of symptom score and viral load (b), and $\log_{10}$ RV16 antibody titers in blood on day 31 after infection (c). At inclusion antibody titers were below 0.78.

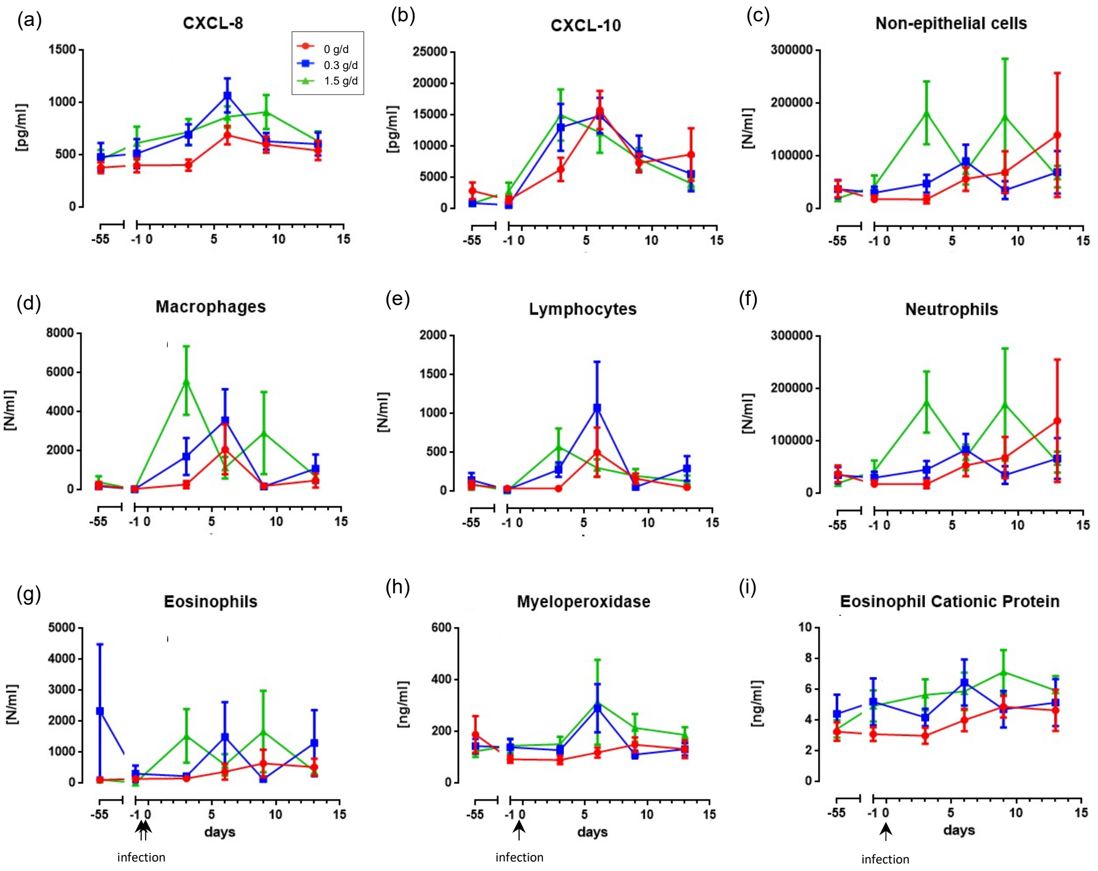

Supplementary figure S2: Absolute values for released selected cytokines (pg per ml) and granular proteins (ng per ml), and cells in nasal lavage fluid (number per ml) in the ITT population at baseline (d-55) just prior to infection (d-1) and during infection (d3, 6, 9 and 13). Each symbol represents the mean ± SEM.

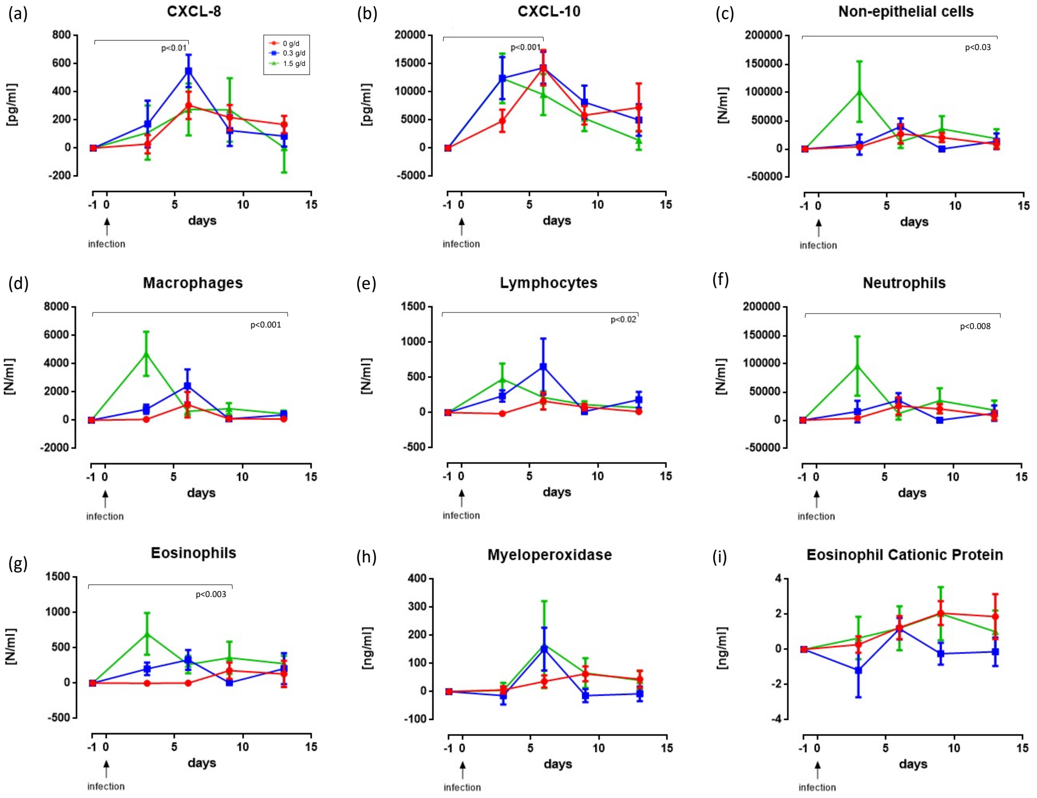

		dose 0 g/d				dose 0.3 g/d				dose 1.5 g/d			
		3	6	9	13	3	6	9	13	3	6	9	13
Non-epithelial cells	ID high	232	**416**	**506***	**506***	**47**	**492**	**376**	**492**	**529**	**38**	**502**	**449**
	value	193 194	688 041	1 758 768	5 399 437	458 450	902 905	695 326	1 585 250	1 908 928	680 675	5 455 293	604 941
	ID low	297		493				**547**	547			161	22
	Value	-166 377		-168 972				-212 438	-393 008			-738 329	-581 833
Macrophages	ID high	545	**416**	386	**506***	**47**	263	**376**	40	231	12	502	**449**
	value	7 958	42 070	2 596	16 232	43 622	56 000	2 933	33 396	48 416	24 361	104 100	104 656
	ID low							**547**					
	Value							-800					
Lymphocytes	ID high	545	**416**	**506***	297	**47**	547	492	40	38	161	44	529
	value	972	13 322	1 761	833	3 043	22 759	789	6 212	7 465	3 978	3 958	3 259
	ID low	387			387								
	Value	-494			-575								
neutrophils	ID high	232	**416**	**506***	506		**492**	**376**	**492**	**529**	**38**	**502**	**449**
	value	190 974	632 649	1 757 007	5 383 205		893 667	6 925 445	1 578 196	1 870 735	675 318	5 351 192	594 476
	ID low	386						**547**	547			161	22
	Value	2 837						-2 119 668	-391 422			-738 329	-581 833
eosinophils	ID high	297	386	386	**506***		**492**		**492**	231	68	449	502
	value	2 716	5 036	13 845	10 821		41 959		38 238	42 202	15 973	65 121	5 296
	ID low	386				**492**		**492**					
	Value	-2 837				-13 151		-8 812					

73 outliers in 24 (of 146) subjects of which one outlier in 6 subjects, two in 5, three in 6, four in 3 and 5, 6,7 and 9 outliuers each in one subject

*506 excluded from PP during blind data review due to excessive use of medication

Supplementary figure S3: For better visualization of time and dose-dependent changes after infection with RV16, changes in soluble markers and cell subsets in nasal lavage provided in Figure 3 are depicted here without the highest and lowest outliers in cell subsets. Subject codes (ID) and values of outliers are summarized in the table. Changes in CXCL-8 (a), CXCL-10 (b), cells in nasal lavage fluid (c-g) and granular proteins (h and i) expressed as change versus value just prior to infection (d-1). Each symbol represents the mean ± SEM. Significance was evaluated by step wise applying the change values of observations per person between d-1 and d13. The reported p-value in the various figures indicates the observed dose-dependent effect over the maximal interval after infection for which significance was observed in the full data set.

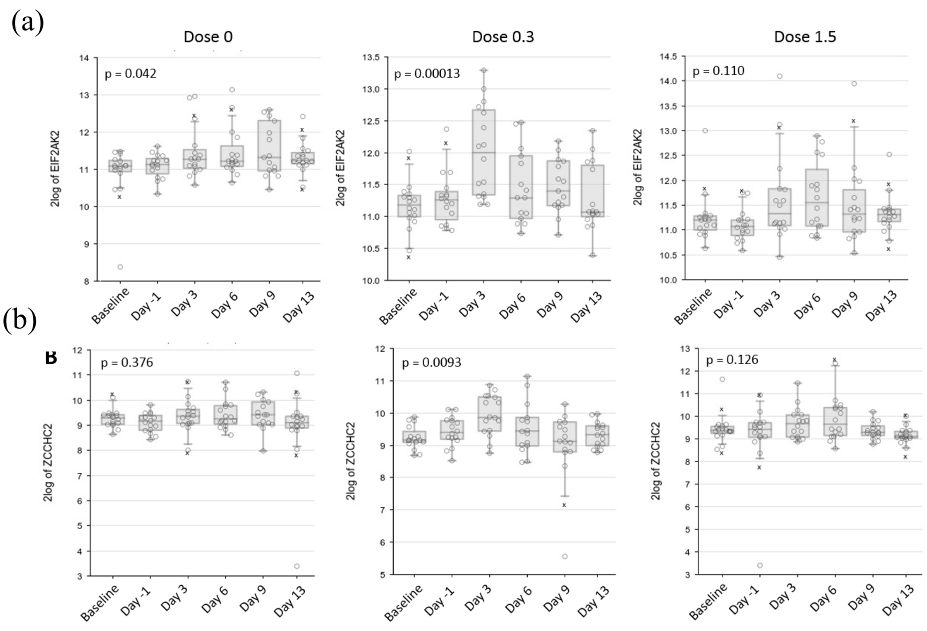

Supplementary figure S4 Expression of the genes *EIF2AK2* (a) and *ZCCHC2* (b) in nasal epithelial cells over time, as a function of treatment.

Supplementary tables

Supplementary table S1. Summary of inclusion and exclusion criteria, dietary restrictions and other relevant criteria

Inclusion criteria

- Age ≥18 and ≤65 years of age;
- Sero-negative (≤ 1:6) to RV16 at screening;
- Body mass index (BMI) ≥ 18.5 and ≤ 30.0 kg/m^2;
- Healthy (assessed by study physician, based on medical history and used medication as provided by the participant);
- Willingness to comply with study procedures;
- Having a GP;
- Signed informed consent.

Exclusion criteria for study enrollment

- History of hay fever and rhinosinusitis;
- History of asthma or COPD;
- History of food allergy;
- Underlying pulmonary, cardiovascular or auto-immune disease;
- History of significant medical or psychiatric disease, at the discretion of the study physician;
- Pregnant or intending to become pregnant during the study period and lactating women;
- Frequent contact with elderly, immune deficient or severe asthma/COPD patients or children under the age of 2 years during the course of the trial;
- NutriLeads, NIZO or AMC employee of departments of Respiratory Medicine and Experimental Immunology;
- Current or ex-smoker (last half year) **;
- Use of statins;
- Consumption of > 14 alcoholic units in a typical week (females) or > 21 alcoholic units in a typical week (males) ***;
- Strenuous exercise (> 10 hrs/week);
- Any other medication at the discretion of the study physician;
- Recreational drug abuse ***;
- Language limitations regarding interviews and questionnaires;
- Volunteers who share the same house(hold);
- Currently participating in another clinical trial;
- Reported, unexplainable weight loss or gain >3 kg in the last month before d-70;
- Night-shift work.

Exclusion criteria for RV16 inoculation:

- Common cold or fever within 7 days prior to RV16 inoculation (assessed by Jackson questionnaire)*;
- A positive respiratory virus PCR, performed 24 h prior to the RV16 inoculation
- Anti-inflammatory drugs or any regular, inhaled or topical nasal drug treatment within 7 days prior to RV16 inoculation;
- Less than 80% compliance with test-product intake;

- Influenza vaccination in the week before RV16 challenge.

* During day -6 till -1 (6 days) of the study period, all subjects were asked to fill in the Jackson questionnaire, once daily, in the evening (at least 1 hour after dinner). Subjects with a common cold or fever up to 1 week before RV-16 inoculation were excluded from the inoculation.

** with 'smoking' we mean, smoking as part of a smoking habit.

*** Abuse: is excessive use or misuse of a substance/alcohol as per physician assessment.

Subjects were asked to continue with the consumption of the test product and to come back for the RV-16 challenge 2 weeks after the original planned date. Re-screening was allowed (but not mandatory) for subjects that received treatment and in the week before RV16 challenge were suffering from a common cold other than a rhinovirus or received influenza vaccination.

A cold was considered present if 2 of the following 3 criteria were present:

- A cumulative symptom score of at least 14 over a 6-day period;
- The subjective impression of a cold (by the volunteer);
- Rhinorrhoea (= nasal drainage/runny nose) on at least 3 days.

Restrictions during the study:

Subjects were requested to continue their habitual diet and refrain from using:

- probiotics
- over the counter medication for common colds
- nasal washes or nose spray
- antihistamines, corticosteroids, and NSAID

Supplementary table S2: Demographic characteristics of enrolled subjects and nested subset

		**Enrolled (N=177)**				**Nested subset (N=58)**				
Parameter	Statistics	0 g/d	0.3 g/d	1.5 g/d	p*	0 g/d	0.3 g/d	1.5 g/d	p*	p#
N		60	58	59		16	16	16		
Age (y)	Mean±SD	39.4 ±16.0	35.2 ±14.3	33.6 ±14.3	0.09	37.4 ±14.8	33.3 ±10.5	32.9 ±14.1	0.57	0.54
Male (%)	N (%)	11 (18)	11 (19)	11 (19)	0.99	3 (19)	3 (19)	3 (19)	1.00	0.96
BMI (kg/m^2)	Mean±SD	23.7 ±2.8	23.5 ±2.9	23.8 ±2.6	0.78	23.9 ±2.4	23.2 ±3.1	23.9 ±2.9	0.72	0.75
Alcohol cons. (glasses/week)	Median (range)	1.5 (0-14)	1 (0-12)	1 (0-10)	0.66	3 (0-10)	0 (0-12)	2 (0-14)	0.04	0.46
Vegetarian diet (N)	Median (range)	0 (0-1)	0 (0-1)	0 (0-1)	0.65	0 (0-1)	0 (0-1)	0 (0-1)	0.48	0.89

* Baseline differences over treatment groups were tested with ANOVA (age and BMI) or Kruskal-Wallis (gender, alcohol use, vegetarian diet)

Comparison to ITT population in table 1, using multiple regression correcting for the doses

Supplementary table S3: Clinical chemistry d-55 compared to d-1

Parameter	Reference range	Day -55						Day -1					
		No dose		Low dose		High dose		No dose		Low dose		High dose	
		mean	sd	mean	sd	mean	sd	mean	sd	mean	sd	mean	sd
**ALAT**	< 45 U/L<br>< 34 U/L	19.3	8.0	22.9	20.8	19.9	7.8	22.7	21.5	19.7	10.1	18.0	8.0
**ASAT**	< 40 U/L	21.8	6.6	23.7	7.8	23.6	12.5	26.0	16.3	24.0	7.3	22.9	10.8
**GGT**	♂:<br><60U/L<br>♀:<40U/L	23.0	29.6	20.7	30.8	17.0	9.9	29.9	62.8	20.1	23.2	16.5	11.5
**Bilirubin**	<17µmol/L	16.8	6.5	17.1	5.50	15.7	5.6	17.8	7.1	16.4	5.1	14.8	5.5

Supplementary table S4: Number of differentially expressed genes in brushed nasal epithelial cells compared to expression just prior to RV16 exposure (d-1) per treatment group.

	Day 3	Day 6	Day 9	Day 13
No dose (0)	7	115	239	1
Low dose (0.3 g/d)	35	622	1	90
High dose (1.5 g/d)	0	2	211	731

Supplementary References

1. Ravi A, Chang M, Pol M, Yang S, Aliprantis A, Thornton B, Carayannopoulos LN, Bautmans A, Robberechts M, Lepeleire ID, et al. Rhinovirus-16 induced temporal interferon responses in nasal epithelium links with viral clearance and symptoms. Clin Exp Allergy 2019;49:1587–97.
